# Loot

**Published:** 1872-02

**Authors:** 


### Loot.

### *Spontaneous Rupture of the Spleen.*

Surgeon F. Odevaine, Bhopal Battalion, reports in *The Indian Medical Gazette* for December 1, 1871, a case of rupture of the spleen occurring spontaneously. The subject was a woman aged 22 years, who just previous to her death had been engaged in grinding corn, and who, it was ascertained positively, had not received a blow in the left hypochondriac region. Feeling faint, she sat down and drank a little water, after which she was able to proceed a little farther, when she was overcome with giddiness and almost immediately died. A post-mortem examination was made. Several pints of serum and clotted blood were found extravasated within the peritoneum. The spleen was about four times its normal size, not remarkably friable, and presented a superficial rent on the middle of its inner aspect about 2½ inches long, and running parallel to its length. The deceased had been subject to epilepsy and ague. The rupture in this case, it will be observed, was on the concave surface or hilus of the spleen, the site of entrance and exit of its numerous very large vessels, and though not very deep, probably in consequence of occupying its most vascular part, fully accounted for the rapid death of this woman, which took place in twenty minutes from the time of her first complaining of giddiness. In cases of ruptured spleen caused by violence, the injury is generally found at the external or anterior edge of the organ.—*Philad. Medical Times.*

***Is Insanity on the Increase?***

Dr. Henry Maudsley, writing on this subject in the *British Med. Journal*, says: "It may be true, and probably is true, that there is more insanity, and more occurring insanity, in proportion to the population now, than at the time when our forefathers followed Boadicea to the battle; but that so many more persons should be yearly going mad now than twenty-five years ago, seems to me a supposition, which is, I will not say preposterous, but is certainly not probable in itself, and not supported by facts. There is abundant evidence of a gradually improved registration of insanity, and of a great increase in the returns in consequence of that improvement, but there is not yet satisfactory evidence of anything more." "Some part of the difference," he says, "is owing to the fact that certain patients are registered as lunatics now who would never have been thought so in times past." Also, "a lower rate of mortality and a lower percentage of recoveries may account for an increase in the total amount of insanity."

***Hesitation in Curing Eczema.***

Mr. Milton (*Med. Press and Circular*) for years has done his best, in every instance, to check the discharge of eczema as quickly as possible. During that period above 5,000 cases have passed under his notice. He concludes that the fear of curing eczema, of however long standing it may be, and however delicate the health of the patient, is not warranted by either proof or analogy; that no known agent possesses the power of repelling eczema; that we can cure it only by means which improve the health at the same time; and that it is as justifiable to arrest its discharge as that of diarrhœa or cholera. All that has been said of eczema may be said of ulcer; there is no danger of healing it up, no bad symptoms ever arose from doing so.—*Medical Record.*

***Impotency.***

Here is a young man, 26 years of age, who is suffering from the result of an early acquired vice—masturbation—a vice which is so degrading in its tendency and injurious in its results that you, as physicians, should ever be ready to raise your voices, calmly and determinedly, against its practice. Its prevention should be your care; its remedy, your study. It is a subject, however, which you must meet wisely, for when a man comes to you with involuntary seminal emissions, it is not then your time to decry the evil. His mind is already diseased (for it is a well-known fact that any disorder about the genital organs is the subject of much solicitude, even though it be perfectly innocent, as varicocele or hydrocele), and it should consequently be your desire to allay his fears and calm his perturbation, since it is often, indeed, that they are perfectly groundless. It is this highly sensitive imagination which renders this class of patients such easy dupes to charlatans, who foster his fears and thereby extort large sums of money.

If his emissions are but few, you can, with truth, assure him that he need not be alarmed, for every healthy celibate man has an occasional emission, in fact, there are instances when vigorous men have had two per week for years without any impairment of health. As a rule, however, they should not occur often, and when frequent should be considered with care.

The first effect of masturbation is to produce an irritable condition of the genital organs themselves, with emissions upon slight excitement; then follow indigestion, palpitation, restlessness, sleeplessness, dizziness, or even partial amaurosis, possibly paralysis, especially paraplegia, and finally the entire disposition is changed, so that the man becomes morose, sullen, distrustful, and desirous of solitude. When he comes to your office he will not look you in the eye, but seems anxious to avoid questions. His mind is constantly fixed upon his unhappy condition, and he is in truth a monomaniac. This is the reason why your treatment should be so greatly directed to the mental condition; in fact, a simple moral control upon your part, with positive and calm reassurance, will often be half the cure.

As a result of the irritable condition of the ejaculatory ducts, there is frequently a viscid discharge from the urethra, which is an alarming symptom to the patient, and renders him still more miserable. This, however, is rarely semen, but is a product of the irritation of the prostate—a form of prostatorrhea. This can be easily diognosed by the microscope from spermatorrhea by the absence of the characteristic spermatozoids. The great weakness and nervous debility are not a result of the direct loss of semen, but of the nerve force.

The treatment, as I have said, must be largely moral; any evil habit, whether it be onanism or excessive venery, must be totally abandoned, and the mind directed away from lasciviousness. The organs are overworked and jaded, and need rest; regular, interesting and varied occupation should be enjoined, for the man must not consider that he is an invalid. Traveling, sea-bathing, and good company will be of great service. The diet should be nutritious but not rich; the supper consisting largely of milk or oysters, with nothing subsequently before retiring. The bladder should be kept well emptied, and any constipation or other irritation of the rectum, as hemorrhoids, carefully removed. The patient should sleep upon his side on a hard mattress, with light covering, having previously employed a cold douche to the spine, followed by vigorous friction. Electricity applied along the course of the genito-crural nerves will also be of much benefit in giving tone and strength to the wasted organs.

Internally I know of no better treatment than phosphorus and strychnia—1-50 of a grain of the former, and 1-30 of the latter—made into a pill, and administered three times daily.

Under this treatment we may hope in this case for a speedy improvement, although he has now been impotent for five months; provided he has moral force enough to let the organs rest.

The use of tinct. cantharid. I consider inadvisable, for it acts but as a spur to a jaded horse. Cauterization of the orifice of the ejaculatory ducts I believe to be but seldom necessary, unless there is great irritability and tenderness at that portion of the urethra.

In regard to the advisability of marriage under circumstances like these, I can only say that the progress of the case must be your guide in giving advice. Fortunately there are many cases that admit of improvement under the influence of matrimony provided moderation be enjoined and the feelings of love engendered. Even though the act be not consummated at first, a little patience and abstinence, combined with good treatment and the encouragement of the surgeon, may at last render its consummation perfect.—*Agnew*, *Clin. Sect.*, *Med. Reporter.*

***Artificial Milk.***

Among the melancholy records of the siege of Paris may be counted those on the utilization of strange materials for food; not the least interesting of these being the researches of M. Boillott, M. Dubrunfant, and M. Charles Fua on "Alimentary Fats."

"Alimentary Fats" is a wide expression, including some rather unsavory hydrocarbons and very curious refuse materials. The main object of these investigations was to determine how such substances may be "usefully employed in alimentation," or, in plain, unsophisticated English, how to make butter from candle ends, dirty drippings, colza oil, fish oils, the refuse of slaughter-houses, the restored grease of the wool-dresser, etc. The general result has proved that the "frying process" is triumphant over its rivals; that by simply raising the temperature of the fat to 140° or 150° centigrade, and in the meantime cautiously sprinkling it with water, the cellular tissue, the volatile oils, the rancidity, offensive odors, and all other non-sentimental impediments to "alimentation" are removable.

This frying process has already effected something like a revolution in the industry of soap-boilers, some of whom have changed their trades to that of butter-fryers.

M. Dubrunfant is not content with superseding the cow in the matter of butter, but has made similar attempts upon milk. He contends that milk is simply an emulsion of neutral fatty matter in a slightly alkaline liquid, such as can be artificially imitated; and that the process of churning consists in hastening the lactic fermentation, thereby acidifying the serum of the milk, and at the same time agglomerating the fatty matter which the acidity sets free from its emulsion. He further controverts the cellular theory by showing that fat-globules of milk do not display any double refraction, as do all organized membranous tissues.

Having thus examined the theoretical constitution of milk, he proceeds to the practical method of imitating it, and gives the following directions :

Add to half a litre of water forty or fifty grammes of saccharine material (cane sugar, glucose, or sugar of milk), twenty or thirty grammes of dry albumen (made from white of egg), and one or two grammes of subcarbonate of soda. These are to be agitated with fifty or sixty grammes of olive oil, or other comestible fatty matter, until they form an emulsion. This may be done with either warm or cold water, but the temperature of 50° to 60° C. is recommended. The result is a pasty liquid, which, by further admixture with its own bulk of water, assumes the consistency and general appearance of milk.

Luxuriously-minded people, who prefer rich cream to ordinary milk, can obtain it by doubling the quantity of fatty matter, and substituting two or three grammes of gelatine for the dry albumen. The researches of Dumas and Fremy having reinstated gelatine among the nitrogenous alimentary materials, M. Dubrunfant prefers it to albumen; it is cheaper, more easily obtained, and the slight viscidity which it gives to the liquid, materially assists the formation and maintenance of the emulsion. By substituting vegetable albumen for the white of egg or gelatine the vegetarian may prepare for himself a milk that will satisfy his uttermost aspirations. —*Nature.—Med. Record.*

### *New Action of the Uterus.*

Dr. F. SEYMOUR inserts this case and remarks in the *Cincinnati Lancet and Observer:*

Mrs. Mary Jane McN——y, residing at Pleasant Ridge, Pendleton county, Kentucky; æt. 32; married at 17 years of age; has been suffering from amenorrhœa from that time until she placed herself under my treatment. She states that she never menstruated since marriage, but at those periods (menstrual) she spat blood and had hemorrhage through the mouth. Had been under medical treatment for sixteen years, under the care of eight different medical men during that time, the majority of whom declared she was incurable. During the time of menstrual periods she suffered, to use her own expression, "death almost." Having previously attended her sister for uterine difficulty to a successful issue, she was prevailed upon to place herself under my care. Upon presenting herself, she appeared like a decrepit woman of fifty years of age, stooped in walking, pale, dejected, nervous, and suffering; her general health almost broken down. Upon examination, found lateral version and anteflexion of uterus, with ulceration of os uteri with granular erosion, the neck congested, indurated, and exquisitely painful. The least touch gave her infinite pain. Used chromic acid, nitrate of silver, and exsiccated sulph. zinc, with pessaries of soda carb., to neutralize the action, and put her under

| | | | |
|---|---|---|---|
| R. | Acid. Hydrocyanic, gttæ., | vj. | |
| | Tinct. Cinchonæ, Comp., | oz. j. | |
| | Tinct. Opii., | dr. ss. | M. |

S.—Coch. parv. i., ter in die sumendum, cum.

| | | |
|---|---|---|
| R. | Zinci Valerianat,<br>Quiniæ Valerianat, | } aa gr. x. |
| Ft. | massa in pil. | xx divid. |

S.—Cap. pil. ij., ter in die altern.

Together with injections of warm water, and cotton steeped in glycerine (glycerole cotton, as it is called,) introduced into the vagina and carefully placed around the cervix uteri. In a short time the ulceration and erosion had healed and the congested and indurated condition of the neck much improved, so much that I was able to try to introduce a uterine probe. Upon attempting to introduce the probe, I found an almost complete stricture of the os internum, through which I could not at first pass a silver uterine probe of the size of a No. ½ catheter. I succeeded, however, in dilating it, and by the aid, first, of sponge and sea-tangle tents, I succeeded in dilating the cervix, and introducing the hysterotome, divided slightly the os internum; after the division, continuing the sea-tangle tent. Her health began to improve, and during the treatment the period of her menstrual flow intervened. To her surprise and gratification, it passed over without any suffering whatever, and a colored fluid (slightly bloody) passed per uterus and vagina. During the three days the treatment was discontinued. As soon as the period was passed, I determined to introduce a galvanic intra-uterine pessary, which I obtained from Mr. Autenreith, and repairing to her house tried to introduce it. I succeeded in introducing it about half an inch, and let her recline on her side, with the intention of pressing it further in. To my astonishment, upon examination to ascertain if it was *in situ*, and to press it up further, I found a peculiar movement of the uterus (vermicular is the best word I can use to describe the peculiar motion), and that the uterus was by its own action drawing up the pessary into itself. Astonished and perplexed, I laid the end of my fore-finger upon the lower part of the cervix uteri, and distinctly felt the shield of the pessary scrape along its palmar aspect, until it not only drew in the entire pessary (2½ inches long), but the shield of the pessary was drawn tight enough to flatten the os uteri by pressure. I asked the patient how she felt, what feelings she had; she said it felt like "pins and needles pricking her," and that "something appeared to be drawing." My apparent astonishment must have alarmed her, as she quickly inquired, in a frightened tone, "Doctor, what is the matter?" Of course, I at once quieted her by an assurance there was nothing. Since it has been introduced, she is much pleased, as it has already done her much good. I examined her to-day (20th), found the pessary still *in situ* perfectly as before, and so well did she appear and so completely established in her health, that I send her home to-morrow (21st), I think, perfectly cured. Has this solved the long-disputed question of impregnation? Has the womb the action of drawing by suction the seminal fluid into its body? I think the seminal fluid is injected into the vagina into the posterior inferior cul-de-sac or curve of vagina, and that the uterus, at the moment of orgasm, while lying in the fluid, by its muscular apparatus suddenly contracts, forcing out the air and creating a vacuum, and by expansion drawing the semen in by suction, as it drew in the pessary, while at the same time it ruptures the ovum in the uterus and lets its contents mingle with the zoosperms in the uterus.

Louis Kossuth communicates to the *Neue Freie Presse* of Vienna the remarkable curative qualities possessed by a grotto near Pistoies, in the valley of Lucques and Pisa, the virtues of which consist in radically curing the gout.

The treatment is easily followed, and lasts from eight to fifteen days.

The patient descends into the grotto, which is well lighted, covered with a bathing-gown. There he has only to sit and admire the stalactites, or converse with his friends. After ten minutes he sweats profusely, but not disagreeably.

After an hour he is taken out, wrapped in a flannel covering, and after reposing a little, subjected to a cold shower-bath.

The curative principle of the grotto, is, however, an enigma.

In the warmest parts of the grotto the air does not show more than thirty-two to thirty-four degrees (centigrades), and is less oppressive than the air outside; the water is still colder, but it is heated by the air, the chemical composition of which resembles that of atmospheric air; the only difference being a slight addition of azote.

Kossuth attributes the remarkable qualities of the grotto to electro-magnetic agents.

The subterraneous grotto in question was discovered some years ago by a number of quarrymen engaged in excavating stone.—*Med. Record.*

***Marriage* v. *Celibacy.***

M. Bertillon read a paper at the sitting of the Academy of Medicine of Paris, on the 14th of November, entitled "Influence du marriage sur la duree de la vie et sur les maladies intellectuelles ou morales," maintaining the healthful influence of conjugal association as compared with that of celibacy, and using as evidence the statistics of France, Holland, and Belgium. According to his figures, between the ages of twenty-five and thirty years 1,000 married men furnish 6 deaths; 1,000 batchelors, 10 deaths; and 1,000 widowers, 22 deaths. From thirty to thirty-five years of age, the same classes, respectively, furnish 7, 11, and 17½ deaths. From thirty-five to forty years of age, the mortality is 7½, 13, and 17½ per 1,000 respectively. And so on in a series of tables for all ages, the married man has always the advantage of the single man; but what is the explanation of the fact that among widowers the mortality is even greater than among bachelors? In the case of men married under the age of twenty, the statistics show anything but a salutary result. Out of 8,000 of this class, the mortality was, before marriage, only 7 per 1,000; *after marriage it was* 50. This is a constant result in Paris, Belgium, and Holland. Everywhere young married people, from eighteen to twenty years of age, die as fast as old people of from 60 to 70 years of age. The conclusion seems evident, according to Hufeland, "that the premature use of the genital organs is the most sure means of inoculating old age." This astonishing mortality being due to no special malady, but to a general enervation, which, without doubt, renders them a prey to any disease.

The same advantage of the marriage state obtains in the case of females, though up to the age of thirty the difference is not so apparent as in the other sex. From thirty to thirty-five the mortality is 11 per 1,000 for single women, and only 9 per 1,000 for married women, and this difference increases up to the age of fifty-five. Thus, from fifty to fifty-five years of age, 1,000 wives furnish only 15 or 16 deaths, whilst as many single women or widows furnish 26 or 27. This advantage remains very notable beyond that age, diminishing but little. In France, however, under twenty-five, and in Paris, under twenty years of age, marriage is far from favorable, but even injurious, as in the case of males. The mortality of unmarried girls of from fifteen to twenty is 7.53 per 1,000; the mortality of wives of the same age being 11.86. The mortality of girls from twenty to twenty-five is 8.32; of wives of the same age, 9.92; the difference in this class being due, no doubt, to pregnancy and its accidents. The deadly effect of widowerhood in men is equally shown in the case of widows up to the age of thirty, and after forty-five, more single women than widows die.

The influence of marriage on criminality is not less striking.

Whilst the criminality of single men for crimes against the person is 100, in married men it is only 49, and 45 for offences against property. The criminality of widows and widowers approaches that of celibacy. Relative to suicide, the author concludes that this cause of death is reduced one-half by marriage.—*Medical Record.*

### *Syrup of the Lacto-Phosphate of Lime.*

The value of phosphate of lime as a restorative remedy in certain forms of imperfect or depraved nutrition, has long been recognized by the profession. A preparation which shall present the phosphate in a soluble and readily assimilable form has been the desideratum. Such a preparation is found in the lacto-phosphate, first recommended by Dr. L. Dusart, in which the lime salt is dissolved in free lactic acid. The lacto-phosphate was employed by Dr. Dusart with favorable results in several cases of tardy union of bones, in rachitis, and in some obstinate cases of diarrhœa and indigestion. The syrup of the lacto-phosphate is prepared by Mr. W. Neergaard, by saturating concentrated lactic acid with the freshly precipitated phosphate of lime, adding for every ounce of lactic acid six ounces and a half of water, an ounce and a half of orange flower water, and twelve ounces of sugar, and digesting in the cold till the sugar is dissolved. The dose for a child is one to two drachms three or four times a day, a drachm of the syrup containing about two grains of phosphate of lime.—*Detroit Review.*

### *Bichlorate of Methylene.*

Samuel Theobald, M.D., of Baltimore, Md., London correspondent of the *Balt. Med. Journ.*, writes that the advantages of bichlorate of methylene as an anæsthetic have been largely tested at Moorfields Hospital, London. No dangerous or unpleasant symptoms caused by its administration have been noticed there. The advantages which it possesses over chloroform are, that it produces its effect more rapidly, and is more evanescent in its action. These properties recommend it more especially for operations of short duration. In its administration, it should be diluted with a much smaller percentage of air than chloroform. To an adult, from one to two drachms may be administered at once, and this will generally prove sufficient, if the operation is of short duration. If desired, however, it may be repeated (four or five times), in drachm doses, as soon as its influence begins to pass off.—*Medical Record.*

